# Comparing the efficacy of EUS versus MRCP with ERCP as gold standard in patients presenting with partial biliary obstruction – finding a better diagnostic tool

**DOI:** 10.12669/pjms.39.5.7280

**Published:** 2023

**Authors:** Rao Saad Ali Khan, Laima Alam, Zoya Ali Khan, Uzair Ali Khan

**Affiliations:** 1 Rao Saad Ali Khan, FCPS Med, FCPS Gastroenterology, FRCP Consultant Gastroenterology and Transplant Hepatologist, Pak Emirates Military Hospital, Rawalpindi Pakistan; 2 Laima Alam, FCPS Gastroenterology, MRCP (UK), CHPE Consultant Gastroenterology, Bahria International Hospital, Rawalpindi Pakistan; 3 Zoya Ali Khan, Research Assistant, Pak Emirates Military Hospital, Rawalpindi Pakistan; 4 Uzair Ali Khan, Research Assistant, Pak Emirates Military Hospital, Rawalpindi Pakistan

**Keywords:** Biliary obstruction, Choledocholithiasis, Endoscopic retrograde cholangio-pancreatography, Endoscopic ultrasound, Magnetic resonance cholangio-pancreatogram

## Abstract

**Objective:**

To evaluate the diagnostic accuracy of different imaging modalities in patients with partial biliary obstruction with no obvious aetiology on initial imaging.

**Methods:**

This is a prospective single-centre cohort study carried out at Pak Emirates Military Hospital, Rawalpindi from June 2019 to June 2021 with non-probability consecutive sampling. Patients with ages 16 to 75 years, presenting with partial biliary obstruction and undetermined aetiology on initial imaging (TUS and MRCP) were enrolled. EUS was performed for each of these patients and the case was regarded as “true positive” or “true negative” if the findings of imaging modality correlated to those of ERCP. ROC curve, sensitivity, specificity, PPV, NPV and AUC (with 95% confidence interval) were drawn for all the diagnostic tools using SPSS V. 21.

**Results:**

A total of 65 patients were enrolled over a period of two years with male to female ratio of 1.4:1. Forty-four patients had an intermediate risk of choledocholithiasis upon preliminary evaluation whereas, 48(74%) of the participants had CBD calculi or sludge confirmed upon subsequent ERCP. Trans-abdominal ultrasound showed the lowest sensitivity (29.2%), specificity (85%), NPV 12% and PPV 93% for diagnosing CBD calculi. This was followed by MRCP with a sensitivity of 37.5%, specificity of 100%, NPV of 36.2% and PPV of 100%. EUS showed the maximum diagnostic accuracy with AUC of 1.0 and a 100% sensitivity and specificity when compared with ERCP as gold standard.

**Conclusion:**

EUS is superior to MRCP in terms of diagnostic accuracy as minimally invasive diagnostic tool and EUS superiority is particularly relevant in patients with intermediate risk of choledocholithiasis.

## INTRODUCTION

Scores of research work and literature is being actively compiled regarding imaging modalities for detecting pancreatico-biliary pathologies with keen interest paid towards cost-effectiveness, diagnostic accuracy, safety and reliability.^1^ Transabdominal ultrasonography is universally employed as the first diagnostic technique for detecting gastrointestinal pathologies but, unfortunately, has low sensitivity for detecting small common bile duct (CBD) stones and sludge^2^ and could achieve definitive diagnosis in only one third of the cases.^3^ Similarly, magnetic retrograde cholangio-pancreatogram (MRCP) has a high sensitivity and specificity for detecting choledocholithiasis and strictures but the sensitivity decreases when stone diameter reaches ≤5mm.^4^

Endoscopic retrograde cholangio-pancreatography (ERCP) is the gold standard tool for detecting CBD pathologies with the added benefit of intervention if required.^5^

Due to its invasive nature, ERCP is rarely preferred as the first choice of investigation and is usually preceded by MRCP or endoscopic ultrasound (EUS).^6^ Endoscopic ultrasound (EUS) is an emerging minimally invasive technique with no risk of radiations or contrast related adverse effects but has limitations such as high cost, non-availability of the facility and trained personnel^7^ and subsequently lack of local database. This study was conducted to evaluate the diagnostic accuracy of different imaging modalities in patients with partial biliary obstruction, presenting either as asymptomatically raised liver enzymes, cholangitis or biliary dilatation with no obvious aetiology on initial imaging. The study helped to establish a better diagnostic tool in patients that pose as diagnostic dilemma with low and intermediate risk for choledocholithiasis.

## METHODS

This was designed as a prospective single-centre cohort study which was performed at Pak Emirates Military Hospital, Rawalpindi from June 2019 to June 2021 after obtaining ethical committee review (A/28/201, dated 1/September/2022) and patients’ consent.

### Patients

The patients enrolled included those with ages 16 to 75 years, presenting with partial biliary obstruction manifested by either unexplained deranged liver function tests (cholestatic picture), cholangitis or dilated CBD (>7mm without cholecystectomy) with undetermined aetiology on initial imaging (TUS and MRCP).^8^ Patients with age less than 16 years, non-consenting individuals, active shock and hemodynamic instability, coagulopathy, severe cardio-pulmonary disease, pregnancy and partial gastrectomy or altered anatomy were excluded.^8^,^9^

All the endoscopic procedures were performed in a dedicated tertiary care advance GI procedure suite by two high volume Consultant Endoscopists with 95% success rate of biliary cannulation, 400 ERCPs per year and performing a regular list of EUS with intervention. Sedation with intravenous midazolam (0.05-0.1 mg/kg) and propofol (0.5-1 mg/kg) was provided to all. Patients with negative EUS were not subjected to further invasive procedures and those with biliary calculi and/or sludge were offered therapeutic ERCP within three days of diagnosis.^10^ The case was regarded as “true positive” or “true negative” if the findings of imaging modality correlated to those of ERCP.

### Statistical analysis

The sampling technique was non-probability consecutive sampling. Qualitative data was represented as frequencies and analysed using Chi square test. Quantitative data was analysed using mean±SD. ROC curve, sensitivity, specificity, PPV, NPV and AOU (with 95% confidence interval) were drawn for all the diagnostic tools namely, trans-abdominal ultrasound, MRCP and EUS with ERCP as a gold standard for reference. All data was analysed using SPSS V.21 with p value <0.05 considered significant.

## RESULTS

A total of 65 patients were enrolled over a period of two years who presented as diagnostic dilemma with normal or mildly raised bilirubin but markedly deranged alkaline phosphatase and/or gamma glutamyl transferase (GGT). Male to female ratio was 1.4:1 with a mean age of 63.4 ± 2.7 years for females and 60 ± 2.4 years for the male participants. Forty-four patients had an intermediate risk of choledocholithiasis upon preliminary evaluation whereas, 48 (74%) of the participants had CBD calculi or sludge confirmed upon subsequent ERCP (Table-I).

**Table-I:** Demographics and ERCP findings of the enrolled cohort (n=65).

Variables	Female	Male
Gender	27(41.5)	38(58.5)
Mean Age (years)	63.4 ± 2.7	60 ± 2.4
Mean ALT (U/L)	61 ± 3.4	60 ± 2.3
Mean AST (U/L)	64 ± 3.5	65 ± 2.5
Mean Bilirubin (umol/L)	25 ± 1.8	29 ± 2.4
Mean Alkaline phosphatase (U/L)	206.3 ± 13.6	219.6 ± 16.6
Mean GGT (U/L)	84 ± 6	91 ± 8.4
Mean TLC (cm^-3^)	8589 ± 353	8452 ± 355
***Choledocholithiasis risk based on initial investigations***		
-Low	1(3.7)	2(5.3)
-Intermediate	19(70.4)	25(66)
-High	7(26)	11(29)
***ERCP findings***		
-CBD stone/sludge	48(74)	
-Upstream CBD dilatation	56(86)	
-Lower CBD stricture	32(49)	
-Normal	4(4.6)	

**Fig1:**
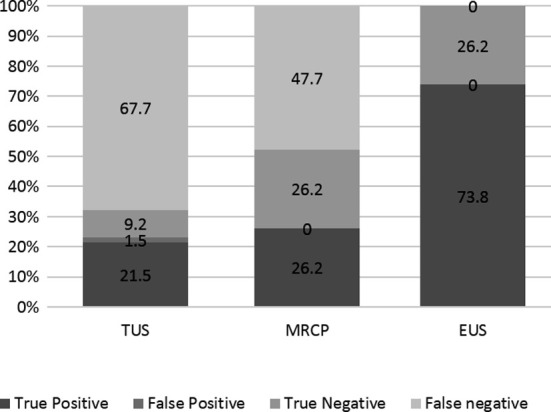
Comparing results of diagnostic modalities with ERCP taken as gold standard.

**Fig.2:**
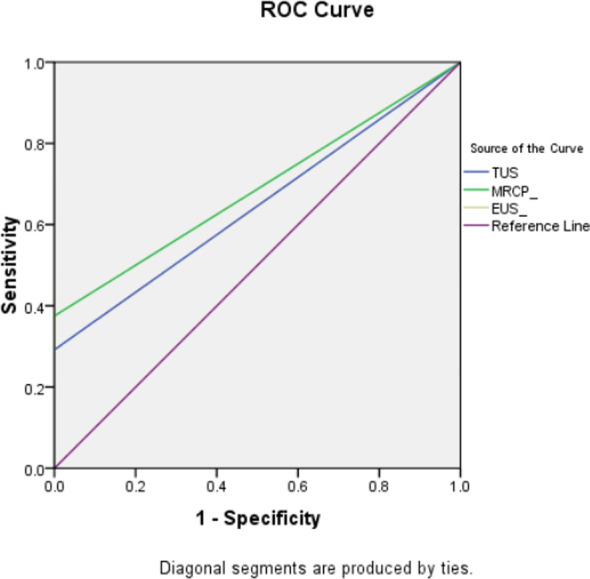
ROC curve for the diagnostic modalities used. Note that AUC for EUS in 1.00.

The diagnostic accuracy and efficacy of the different modalities utilized in evaluating partial biliary obstruction are summarized in Table-II & III and (Fig.1). Trans-abdominal ultrasound showed the lowest sensitivity (29.2%), specificity (85%), NPV 12% and PPV 93% for diagnosing CBD calculi. This was followed by MRCP with a sensitivity of 37.5%, specificity of 100%, NPV of 36.2% and PPV of 100%. EUS showed the maximum diagnostic accuracy with AUC of 1.0 (Fig.2) and a 100% sensitivity and specificity when compared with ERCP as gold standard.

**Table-II:** Comparing results of imaging modalities with ERCP taken as gold standard.

Imaging modality			ERCP findings		p-value (Chi square statistics)

			Calculi/sludge	Negative	
TUS	Calculi/sludge	15	14	1	0.014
	Negative	50	44	6	
MRCP	Calculi/sludge	17	17	0	0.002
	Negative	48	31	17	
EUS	Calculi/sludge	48	48	0	≤0.001
	Negative	17	0	17	

**Table-III:** Efficacy of different imaging modalities in comparison with ERCP taken as gold standard.

Indicators	TUS	MRCP	EUS
Area Under Curve (AUC)	0.646	0.688	1.00
Negative Predictive Value (NPV)	12%	36.2%	100%
Positive Predictive Value (PPV)	93%	100%	100%
Sensitivity	29.2%	37.5%	100%
Specificity	85%	100%	100%

## DISCUSSION

With advancements in healthcare provision, the stacks are aimed at cost effective minimally invasive procedures with high efficacy, reproducibility and safety. Although ERCP has emerged as a gold standard for diagnosing and treating pancreatico-biliary diseases, it is dependent on operator expertise, intra and post procedure complications and the availability of a dedicated hepato-biliary unit and a multi-disciplinary team.^11^

Our study aimed at those patients that presented as a diagnostic dilemma to our tertiary care facility with deranged alkaline phosphatase and/or gamma glutamyl transferase on the background of normal or near normal bilirubin and indeterminate aetiology on either trans-abdominal ultrasound, MRCP or both. There is plenty of literature available regarding the diagnostic accuracy of different tools in case of choledocholithiasis but none was available for partially obstructed biliary channels and microlithiasis/sludge.

This study showed that TUS had a good specificity (85%) and PPV (93%) but a very low sensitivity (29.2%) and NPV (12%) for diagnosing partial biliary obstruction. This implies that the number of false negatives for TUS was high for our cohort. Other studies looking in to the diagnostic accuracy of ultrasound for detecting choledocholithiasis also reported a low sensitivity (15-40%).^9^ The reasons for the low sensitivity outlined in the literature are subjective nature of this tool, difficulty in differentiating air bubbles from other acoustic shadows (as in cholangitis), interference from the abdominal fat and bowel gas shadows and the inability to pick up smaller stones.^12^

MRCP findings for our study had a relatively better sensitivity (37.5%) and NPV (36.2%) as compared to TUS and a 100% specificity and PPV when compared to ERCP. This sensitivity and NPV was lower than other studies where all the cohort had deranged LFTs and were symptomtic.^9^,^11^ Another study with 90 suspected choledocholithiasis patients, all of whom had symptoms and deranged LFTs showed a specificity of 87.5% and a PPV of 94.7%.^13^ This phenomenon was also studied by other researchers where the diagnostic accuracy was affected by the presence of symptoms and abnormal liver enzymes^1^,^14^-^16^, the reason why the accuracy was only marginal for our cohort with normal or near-normal bilirubin (partial biliary obstruction).

EUS has emerged as an invaluable asset in every sophisticated GI procedure suite with its minimally invasive approach, acceptable safety profile and a high diagnostic accuracy. Our study showed a 100% sensitivity and specificity for EUS in comparison to ERCP as the gold standard which was in accordance to the pooled sensitivity and specificity.^15^ EUS does not employ radiations or contrast media and can be safely used in patients with embedded metal fragments/devices.

Although literature reports similar diagnostic efficacy for MRCP and EUS for detecting CBD pathologies (77-100% sensitivity and 70-99% specificity)^16^, the performance of MRCP declines for microlithiasis (<5mm), dilated CBD and smaller ampullary lesions.^17^ Also, the inability to take tissue samples, the chances of claustrophobia, technical difficulty in jittery/tremulous patients and the use of contrast make MRCP unsuitable for high risk patients.^18^ EUS on the contrary can pick up diminutive stones and can be used for histological diagnosis.^19^

A cost-analysis study from Thailand concluded that EUS preceding ERCP procedure was more cost-effective as EUS could successfully rule out CBD stones, eliminating the need for invasive ERCP.^7^ The same study also reported a 60%-70% less cost of EUS versus diagnostic and/or therapeutic ERCP in lieu of lesser post-ERCP complications, hospital stay, rehabilitation and work days lost. A study by Benjaminov et al found that performing EUS and ERCP in the same session were safer than postponing ERCP as that led to biliary complications.^20^ This approach cannot be employed for resource limited set-ups where majority of the population is living below the poverty line.^21^

### Limitations

It is a single-centre data and paucity of local data to compare results with. Also cost effectiveness, procedure related complications and tissue diagnosis were not taken in to consideration.

## CONCLUSION

In conclusion, EUS is superior to MRCP in terms of diagnostic accuracy as a minimally invasive diagnostic tool and EUS superiority is particularly relevant in patients with intermediate risk of choledocholithiasis.

### Authors’ contribution:

**RSAK:** Contributed to the idea, data collection, patient care and critical review.

**LA:** Contributed to the study design, pro forma, statistical analysis and drafting of the manuscript.

**ZAK, UAK:** Contributed to data collection.

All the authors take equal responsibility for the accuracy and integrity of the work.
